# Ocular Risk Factors for Exudative AMD: A Novel Semiautomated Grading System

**DOI:** 10.1155/2013/464218

**Published:** 2013-07-30

**Authors:** João Pedro Marques, Miguel Costa, Pedro Melo, Carlos Manta Oliveira, Isabel Pires, Maria Luz Cachulo, João Figueira, Rufino Silva

**Affiliations:** ^1^Centro Hospitalar e Universitário de Coimbra (CHUC), 3000-075 Coimbra, Portugal; ^2^Association for Innovation and Biomedical Research on Light and Image (AIBILI), 3000-548 Coimbra, Portugal; ^3^Critical Health SA, 3045-504 Coimbra, Portugal; ^4^Faculty of Medicine, 3004-504 Coimbra, Portugal

## Abstract

*Purpose*. To evaluate the contribution of the ocular risk factors in the conversion of the fellow eye of patients with unilateral exudative AMD, using a novel semiautomated grading system. *Materials and Methods*. Single-center, retrospective study including 89 consecutive patients with unilateral exudative AMD and ≥3 years of followup. Baseline color fundus photographs were graded using an innovative grading software, RetmarkerAMD (Critical Health SA). *Results*. The follow-up period was 60.9 ± 31.3 months. The occurrence of CNV was confirmed in 42 eyes (47.2%). The cumulative incidence of CNV was 23.6% at 2 years, 33.7% at 3 years, 39.3% at 5 years, and 47.2% at 10 years, with a mean annual incidence of 12.0% (95% CI = 0.088–0.162). The absolute number of drusen in the central 1000 and 3000 **μ**m (*P* < 0.05) and the absolute number of drusen ≥125 *µ*m in the central 3000 and 6000 *µ*m (*P* < 0.05) proved to be significant risk factors for CNV. *Conclusion*. The use of quantitative variables in the determination of the OR of developing CNV allowed the establishment of significant risk factors for neovascularization. The long follow-up period and the innovative methodology reinforce the value of our results. This trial is registered with ClinicalTrials.gov NCT00801541.

## 1. Introduction

Age-related macular degeneration (AMD) poses a substantial public health problem worldwide [1–4]. The exudative form is the leading cause of irreversible vision loss in subjects over 65 years of age living in economically developed countries and accounts for >80% of cases of legal blindness associated with the disease [5–7]. Risk estimation is crucial in order to provide adequate patient monitoring. Fellow eyes of individuals with unilateral exudative AMD should receive the utmost attention since they are known to have an increased risk of developing choroidal neovascularization (CNV) [4, 5, 8, 9].

The role of retinal imaging in AMD has long been recognized. Several studies have concluded that digital imaging is reliable for the purpose of grading AMD, both in clinical practice and in clinical trials [10, 11]. The International Classification and Grading System [12] was created to provide a uniform and internationally accepted nomenclature and grading system for the disease, based on color fundus photography's morphological findings. Implementation of this system in large cohort epidemiological studies helped establishing significant determinants for the development of wet AMD [4, 13–15] and led to the creation of a fundus photography-based severity scale by the AREDS group [16]. This simplified scale uses a point scoring system and estimates the 5-year risk of developing advanced AMD. The three variables included are the presence/absence of (1) large drusen (≥125 *μ*m), (2) pigmentary changes (hypo- or hyperpigmentation) and (3) neovascular AMD. The major goals of risk estimation are to provide baseline risk categories, to allow tracking of progression, and to define surrogate outcomes for progression to the advanced forms of the disease [17]. 

The importance of drusen characteristics (such as the area and number of drusen) in the development of wet AMD has been highlighted by several studies [13–15, 18]. When grading is performed with conventional reading center techniques, the depiction of these variables is categorical in nature. Taking the number of drusen as an example, a simplified categorical assessment, as provided by the International Classification and Grading System for AMD [12], would grade one image having 0, 1–9, 10–19, or ≥20 drusen. Statistical analysis models using continuous variables (such as the real number of drusen or the real area of drusen) are generally more robust, hence providing far more reliable results [19]. 

The advent of computer-assisted grading systems represents a significant advance in retinal imaging. Not only do they make the grading process more efficient, but they also deliver faster data analysis when compared to the more labor-intensive conventional reading center methods [18, 20–22]. 

Using a novel semiautomated grading software capable of categorical and continuous variable grouping (RetmarkerAMD), this study aims to evaluate the contribution of the ocular risk factors in the development of CNV in the fellow eyes of patients with unilateral exudative AMD.

## 2. Material and Methods

The retrospective, single-center study included 89 consecutive patients with unilateral exudative AMD. Patients with neovascular AMD in one eye (the nonstudy eye) and early age-related maculopathy in the fellow eye (study eye) were enrolled. Inclusion criteria were (a) age ≥ 50 years; (b) any race and either sex; (c) clinical diagnosis of wet AMD in one eye (non-study eye), and the presence of the following characteristics in the second eye (study eye): (1) ≥5 intermediate drusen (≥63 and <125 *μ*m), ≥1 large soft drusen ≥ 125 *μ*m, and/or confluent drusen, within the central 3,000 *μ*m region, and (2) with or without pigmentary changes; (d) ≥3 years of followup after development of CNV in the non-study eye; and (e) baseline color fundus pictures of the study eye at the time of conversion of the non-study eye. Exclusion criteria were (a) presence of other ophthalmologic diseases likely to jeopardize the grading of the color fundus pictures; (b) non-AMD-related CNV in the non-study eye; (c) clinical or fundoscopic signs of myopic retinopathy or refractive power ≥8D; and (d) evidence of past or present CNV in the study eye.

### 2.1. Grading Fundus Color Photography

Mydriatic color fundus digital photographs taken at the time of conversion of the non-study eye were used for grading. The pictures were centered on the fovea, with a 30-degree field of view. Grading took place at the Coimbra Ophthalmology Reading Center (CORC) by graders certified by this institution. For the certification process, an agreement between graders was developed as generalized kappa-type statistics, with a kappa value >0.61 required for approval [23].

Grading was performed using the RetmarkerAMD (Critical Health SA), a computer-assisted grading system that follows the guidelines of the International Classification and Grading System for AMD [12]. The system does not automatically detect lesions, but its components allow to (i) differentiate graded from nongraded images; (ii) assess images in full-screen view; (iii) draw free forms of all lesion types over the images; (iv) draw preset circular objects of different color codes and sizes (63 *μ*m, 125 *μ*m, 175 *μ*m, 250 *μ*m, or 500 *μ*m) to represent drusen, hyperpigmentation, hypopigmentation, geographic atrophy, and exudative lesions; (v) mark drusen as confluent; (vi) zoom in/out the image or display an image area with a higher/lower degree of magnification; (vii) measure distances in the image and control red/blue/green channels (RGB), brightness, and contrast; (viii) superimpose a standard grid to identify eye subfields, and (ix) manually enter patient data.

**Figure 1:**
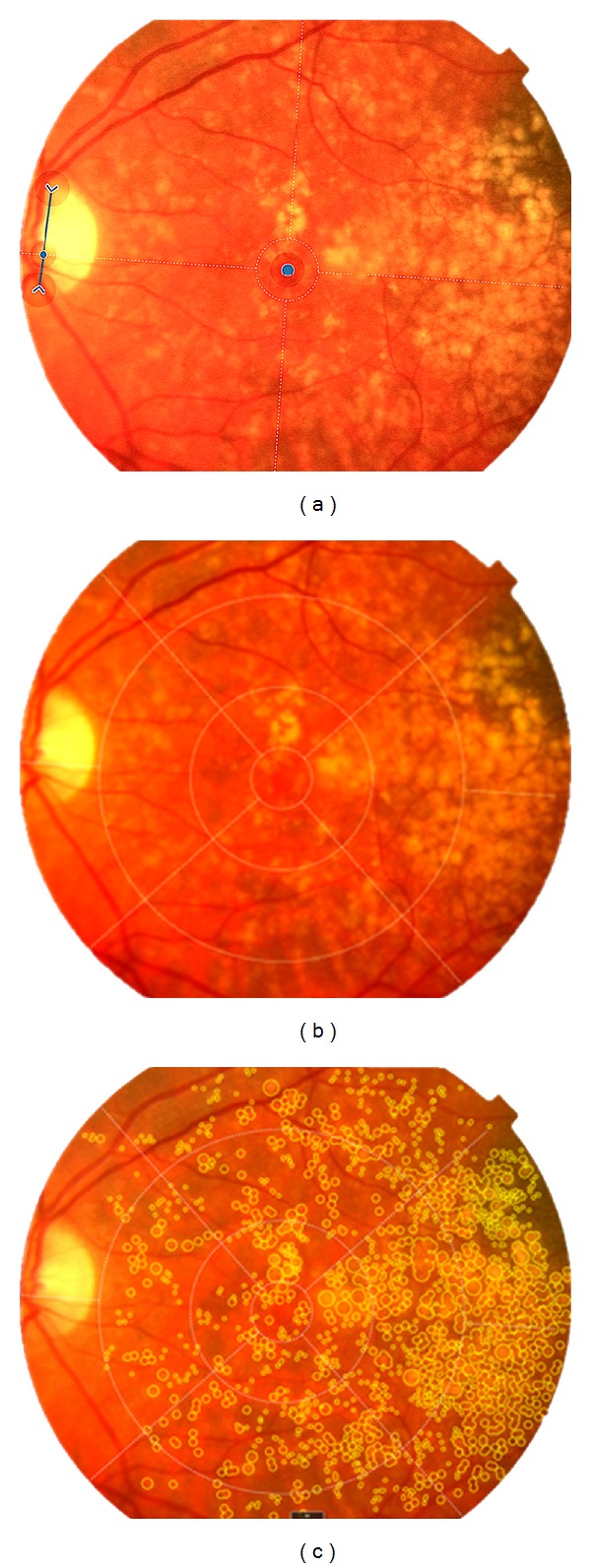
Print screens of real-time grading using RetmarkerAMD. (a) Calibration is achieved after manually identifying the fovea and establishing optic disc diameter (blue dot and arrow, resp.). The software then generates a reference grid (b) that follows the International Classification and Grading System for AMD [12]. (c) Both free forms and predefined circles (63 *μ*m, 125 *μ*m, 175 *μ*m, 250 *μ*m, and 500 *μ*m) can be used for quantifying fundus features in digital color fundus photographs, including drusen, pigmentary changes, geographic atrophy, or exudative lesions.

Each image is calibrated before any measurement is performed. The user manually indicates the optic disc vertical limits along with the center of the macula (fovea), as shown in Figure 1(a). An optic disc with diameter of 1.5 mm is used as reference by the software to set up an overlaying reference grid (diameter 6000 *μ*m), where measurements take place. The grid consists of 3 concentric circles and a right-angled cross at 45° and 135° to the horizontal, which is adjusted according to the previous calibration (Figure 1(b)). As per the International Classification and Grading System [12], the diameters of the central, inner, and outer circles are 1000, 3000, and 6000 *μ*m, respectively. 

The fundus signs allowed to be graded by this tool include (a) number of drusen (total, <63 *μ*m, ≥63 and <125 *μ*m, and ≥125 *μ*m); (b) drusen type (hard, soft distinct, soft indistinct, semisolid, serogranular, or crystalline); (c) total area occupied by drusen (<1%, <10%, <25%, <50%, ≥50%, for central, inner, and outer circles separately); (d) cumulative real area occupied by drusen and area of each subfield; (e) confluence of drusen (absent, <10%, <50%, ≥50%); (f) hyperpigmentation and hypopigmentation (absent, <125 *μ*m, <175 *μ*m, ≥175 *μ*m) of the retinal pigment epithelium; (g) atrophic AMD; and (h) neovascular AMD. 

Using this semi-automated grading system, the results are displayed on screen in real time, and the final characterization of soft distinct, indistinct, reticular, or crystalline drusen is performed by the operator. 

Preliminary results from comparative tests held at our institution have shown that RetmarkerAMD is more effective than 35 mm film manual grading [20]. Aside from being timesaving (overall 35% faster), the software has also proved to be more accurate (overall 32% more lesions identified) [Silva R., oral presentation, EURETINA 2010]. 

### 2.2. Statistical Analysis

Data were characterized with descriptive statistics and explored with classification trees using CRT algorithm. Differences between eyes that converted and those who did not were assessed using the Pearson *χ* ^2^ test and Fisher's exact test for nominal variables and the Student's* t*-test and Mann-Whitney *U* test for scalar variables. Cumulative hazards for conversion times were calculated. Multivariate logistic regressions were used to calculate the odds ratio, expressing the effects of all of the tested explanatory factors for eye conversion in two models: (1) considering only large drusen (≥125 *µ*m) and (2) considering all drusen. The following parameters were considered in the multivariate analysis: real number of drusen, real area occupied by drusen, presence of hyperpigmentation, and age at diagnosis. Results with *P* values <0.05 were considered statistically significant. Data were analyzed using STATA software (StataCorp, College Station, TX, USA) Version 12.1 SE and SPSS 16.0 program (Statistical Package for Social Sciences; SPSS Inc., Chicago, IL, USA).

## 3. Results

Eighty-nine subjects were enrolled in the study. The study eye was the right eye in 47 individuals (52.8%) and the left eye in 42 (47.2%) individuals. The patients were aged between 55 and 92 years (mean ± SD: 74.2 ± 6.86 years), with a slight female predominance—58.4% (*n* = 52). The average age at conversion of the study eye was 77.5 ± 6.16, years and the time to conversion ranged between 12 and 113 months (median 28.5; IQR 24–43). The follow-up period was 60.9 ± 31.3 months (median 37; IQR 36–74).

**Figure 2:**
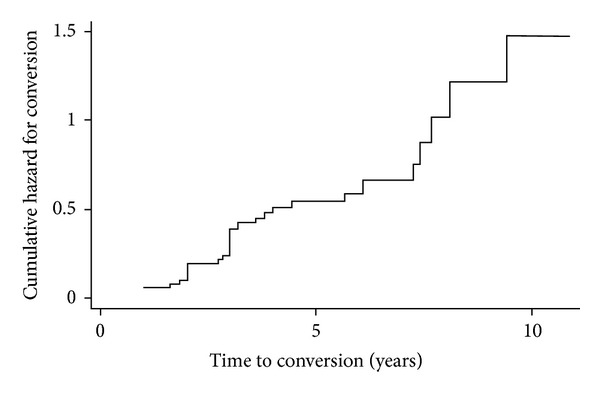
Cumulative hazard for conversion over the ten-year followup period.

The occurrence of CNV was confirmed in 42 (47.2%) eyes during followup (Table 1). The progression of the cumulative risk of conversion is shown in Figure 2. The incidence of CNV, calculated for the follow-up period, was 12.0%/year (95% CI = 0.088–0.162).

Even though drusen were universally identified (*n* = 89), pigmentary changes were only found in a small group of patients (*n* = 22). The variables initially tested to determine the predictive value of conversion in exudative AMD are shown in Table 2. In this first approach, we built the univariate analysis system using categorical variables, exported as such from the RetmarkerAMD software: number of drusen (0, 1–9, 0–19, ≥20), area occupied by drusen (<1% <10%, <25%, <50%, ≥50%), hypo- and hyperpigmentation (absent, <63 *μ*m, ≥63 *μ*m), and confluence of drusen (<10%, <50%, ≥50%). Drusen area within the inner circle was the only variable demonstrating a statistically significant different distribution across the conversion group (*P* < 0.05).

In order to increase the robustness of the analysis, we used continuous variables: the real (absolute) number and area occupied by drusen (Table 3). We tested these variables for the totality of drusen and for large drusen (≥125 *μ*m) only and separately for the central 1000 *μ*m, 3000 *μ*m, and 6000 *μ*m in order to appraise the role of the location in the development of neovascular events. Results are shown in Table 4. The real number of drusen in the central 1000 *μ*m was the only variable with a statistically significant result (*P* < 0.05).

Multivariate logistic regressions were then conducted, calculating the odds ratio (OR) for each covariate. The parameters considered were real number of drusen, real area of drusen, presence of pigmentary changes, and age at diagnosis. We defined two models: (1) considering large drusen (≥125 *μ*m) only and (2) considering the totality of drusen. The results obtained for the two models tested are shown in Tables 5 and 6, respectively. In the first model, the increase in number of drusen ≥ 125 *μ*m showed a positive effect on the risk of conversion in the central 3000 and 6000 *μ*m regions. The increase in the area occupied by drusen ≥ 125 *μ*m and the presence of hyperpigmentation resulted in a decreased risk of developing CNV. In Model 2, increases in the total drusen number showed a positive effect on the risk of conversion in the central 1000 and 3000 *μ*m regions. The presence of hyperpigmentation showed to decrease the risk of CNV in the central 3000 and 6000 *μ*m regions.

## 4. Discussion

Prior studies (MPS [14, 15], AREDS [16]) comprising grading and risk estimation based on morphological assessments of baseline color fundus photography used manual grading and had their grading data grouped according to the guidelines of the International Classification and Grading System for AMD [12]. As such, depiction of variables such as the number (0, 1–9, 0–19, ≥20) and area occupied by drusen (<1% <10%, <25%, <50%, ≥50%) was categorical in nature, which inevitably produces some estimates that can be a source of bias. 

Although still not widely used in clinical or research settings, the development of semi-automated image analysis systems can significantly reduce the time and expense involved in manual grading. The use of innovative software in digital color fundus photography grading is changing the way reading centers work around the world, as it represents a convenient and versatile way of imaging and grading fundus changes. Friberg et al. [19] used a computer-based algorithm to detect and characterize drusen on digitized images and showed that quantitative detection of drusen can be performed reproducibly and efficiently using this method [19]. When compared to more labor intensive reading center techniques, the results were similar but the algorithm showed higher precision and accuracy [19]. 

RetmarkerAMD is a revolutionary platform with a user-friendly interface built upon the International Classification and Grading System for AMD's guidelines [12]. The software has already been validated for quantification of AMD features [Simão, S. et al., poster presentation, ARVO 2011] and is currently being used in an ongoing epidemiologic study of AMD in the Portuguese population with an estimated number of 4000 participants (NCT01298674). When compared to manual grading, the software was more effective and less time consuming (nearly 35% faster), allowing identification of 32% more lesions and reducing human error [Silva R., oral presentation, EURETINA 2010]. One must not forget, however, that, even though the advantages of using computer-assisted grading systems are many, it may also be a source of bias. One example would be image calibration with the generation of the overlaying grid based on a reference optic disc with a diameter of 1.5 mm. Although this estimation of the optic disc diameter has been used in several studies, it does not account for the few cases where anatomic variants of the optic disc are present and may thus constitute a weakness of the software. 

RetmarkerAMD produces standardized and detailed records in Excel that allows easy data mining and statistical analysis. Variables can be grouped both in a categorical and in a continuous manner. Since statistical models operating with quantitative variables provide more reliable results [19], we took advantage of this unique ability to explore the effect of drusen characteristics in the conversion of the fellow eye. Our study combines the advantages of this innovative and validated semi-automated software with a long-term followup of a cohort consisting purely of fellow eyes of patients with unilateral AMD. With this approach we meant to eliminate the most consistent risk factor for CNV [17, 18], consequently enabling better understanding of the role of drusen characteristics in the development of wet AMD. 

When testing the variables in a categorical manner, the total area occupied by drusen within the inner circle (central 3000 *μ*m) was the only variable associated with the development of CNV (*P* < 0.05). This finding is consistent with the results of the AREDS group [16], where variables were tested categorically, and drusen area appeared to be a stronger and more consistent risk factor than the number of drusen. Interestingly, however, when we used continuous variables, namely, the real (absolute) number and area of drusen, the influence of drusen area in the conversion lost its statistical significance, and the real number of drusen in the central 1000 *μ*m became the only variable correlated with the development of exudative AMD in the fellow eye (*P* < 0.05).

In order to clarify the role of each determinant independently, we conducted multivariate logistic regressions and calculated the odds ratio (OR) for each covariate. We found that both the real number of (total) drusen (in the central 1000 and 3000 *μ*m) and the real number of (large) drusen ≥ 125 *μ*m (in the central 3000 and 6000 *μ*m) were significant risk factors for the occurrence of neovascular events (*P* < 0.05). However, the real area occupied by drusen ≥ 125 *μ*m proved to be inversely correlated with the development of CNV (*P* < 0.05). The AREDS group [16] stated that the number of large drusen (≥125 *μ*m) is closely related to the total area of drusen and that the latter is a significant risk factor for wet AMD. In a study conducted by Friberg et al. [21], aiming to determine the relationship between the number of drusen and drusen area, the authors found that the number of large drusen does not correlate better with total drusen area than drusen of other sizes, meaning that the number of large drusen is not necessarily a good surrogate for total drusen area. The same authors went further in another study [18] where digital images of 949 eyes of patients included in AREDS [16] and PTAMD [28] studies were retrospectively evaluated by computerized methods, and the variables were analyzed in a continuous manner. The author concluded that total drusen area was not a consistent risk factor for the development of CNV, and although the probability of a neovascular event would increase with increasing drusen area initially, the risk would then invert as a total drusen area of ~0.75 mm^2^ was reached (~60 large drusen) [18]. This finding might justify our results. One possible explanation would be that the eyes with higher drusen area develop geographic atrophy (GA) instead of CNV. In CAPT [29], even though there was no association between any of the drusen measurements and the development of CNV, a correlation was found between drusen area and the development of GA.

Like drusen size and area, pigmentary changes have also been recognized previously as a risk factor for progression to wet AMD, both in individuals free of advanced AMD bilaterally and in the fellow eye of subjects with unilateral exudative AMD [15, 16, 29]. The presence of hyperpigmentation is one of the risk factors associated with CNV determined by the MPS group [15] and is also part of the AREDS simplified severity scale [17]. In our study, the presence of hyperpigmentation was inversely correlated with the development of exudative AMD in both models of multivariate analysis. This finding was unexpected. Friberg et al. [18] found that this determinant was not a consistent risk factor for an eye's development of CNV, as the location of the hyperpigmentation would influence its effect. In CAPT [29], hyperpigmentation was associated both with CNV and GA. As it represents degeneration of the retinal pigment epithelium (RPE) and a relatively advanced stage of drusen evolution, it is possible that either CNV or GA can follow its appearance. Since we have not looked for the development of GA in our cohort, we cannot correlate both. The difficulties detecting hyperpigmentation and the relatively few patients where we found it (*n* = 22) may have influenced our results. 

The incidence of CNV in the fellow eyes of subjects with unilateral exudative AMD is known to be high. In our study there was a conversion rate of 12%/year (95% CI 0.088 to 0.162), a result consistent with other studies where it ranges from 6 to 12%/year [15, 25, 30–32]. 

From the 89 patients enrolled, 42 (47.2%) developed CNV during the follow-up period (an average of 5.1 years). This number is higher than the ones reported by the extrafoveal trial of the MPS group [14] (25.7% in a cohort of 128 eyes with a 5-year followup) or the SST group [25] (26.9% in a cohort of 364 eyes with a 4-year followup). The long-term followup of our study (ranging from 36 to 148 months) may justify some of this discrepancy. When comparing our results with the AREDS subgroup with unilateral exudative AMD, where the mean followup was 6.3 years, the disparity is less prominent (38.9% in a cohort of 714 eyes). 

The effect of systemic (smoking history, hypertension, body mass index, and dietary habits) or genetic factors in the conversion of the fellow eye was not accounted for in our study. Although these might have had some impact, apart from the smoking history, the influence of these determinants in the development of exudative AMD is substantially less important than the contribution of the ocular risk factors [13].

The cumulative incidence of CNV was 7.9% at the 1st year, 23.6% at 2 years, 33.7% at 3 years, 39.3% at 5 years, and 47.2% at 10 years. These results are identical to those identified in other large cohort studies [14, 15, 24–27], summarized in Table 7. Opposing to the studies of Caucasian populations, it is noteworthy to sign that, among Asians [27], the cumulative incidence of CNV in the fellow eye is considerably lower.

In conclusion, RetmarkerAMD allowed the use of quantitative variables in the determination of the OR of developing CNV, thus establishing the real (absolute) number of drusen (in the central 1000 and 3000 *μ*m) and the real number of drusen ≥ 125 *μ*m (in the central 3000 and 6000 *μ*m) as significant risk factors for the conversion of the fellow eye. The long follow-up period and the use of an innovative and validated semi-automated grading software reinforce the value of our results and support the influence of the ocular risk factors in the progression of the fellow eye to exudative AMD.

## Figures and Tables

**Table 1:** Choroidal neovascularization: number or events, cumulative incidence, and cumulative risk over the ten-year period of follow up.

Time (years)	CNV occurrence (*n*)	Cumulative incidence (%)	Cumulative risk
1	7	7.9	0.0787 (95% CI = 0.0375–0.1650)
2	14	23.6	0.2532 (95% CI = 0.1650–0.3885)
3	9	33.7	0.3889 (95% CI = 0.2713–0.5575)
5	5	39.3	0.5418 (95% CI = 0.3787–0.7750)
10	7	47.2	1.4674 (95% CI = 0.8547–2.5194)

**Table 2:** Univariate analysis to determine the predictive value of conversion, using variables expressed in a categorical manner.

Variable	Pearson *χ* ^2^	*P* value
Predominant type of drusen in the central 6000 *μ*m	—	0.697
Number of drusen	—	0.535
Area covered by drusen in the central 1000 *μ*m	—	0.805
Hyperpigmentation	—	0.515
Hypopigmentation	—	0.192
Number of drusen <63 *μ*m	—	0.566
Number of drusen ≥63 *μ*m and <125 *μ*m	6.5333	0.088
Number of drusen ≥125 *μ*m	—	0.848
Total area covered by drusen	—	0.358
Total area covered by drusen in the central 3000 *μ*m	—	**0.024**
Total area covered by drusen in the central 6000 *μ*m	—	0.470
Confluence of drusen	—	0.738
Age-related macular degeneration stage	—	0.604

**Table 3:** Baseline morphologic drusen characteristics (number and area) expressed as continuous variables.

Parameter	*n*	Median	Interquartile range
*Within central 1000 *μ*m *			
Number of drusen ≥125 *μ*m	89	1	0–4
Total number of drusen	89	6	1–13
Area occupied by drusen ≥125 *μ*m	89	12	0–83
Total drusen area	89	44	7–113

*Within central 3000 *μ*m *			
Number of drusen ≥125 *μ*m	89	26	1–47
Total number of drusen	89	83	33–143
Area occupied by drusen ≥125 *μ*m	89	351	12–966
Total drusen area	89	689	127–1322

*Within central 6000 *μ*m *			
Number of drusen ≥125 *μ*m	89	54	10–132
Total number of drusen	89	123	38–210
Area occupied by drusen ≥125 *μ*m	89	742	93–1973
Total drusen area	89	1329	404–3180

**Table 4:** Univariate analysis to determine the predictive value of conversion, using continuous variables.

Parameter	Mann-Whitney *U* test (*P* value)	
	Drusen ≥ 125 *μ*m	All drusen
*Central 1000 *μ*m region *		
Real number of drusen	0.3232	**0.0308**
Real area of drusen	0.6274	0.3118

*Central 3000 *μ*m region *		
Real number of drusen	0.0881	0.0844
Real area of drusen	0.4725	0.5126

*Central 6000 *μ*m region *		
Real number of drusen	0.5287	0.3785
Real area of drusen	0.7529	0.6870

**Table 5:** Model 1 of multivariate analysis: logistic regression with odds ratio (OR) estimates considering only large drusen (≥125 *μ*m).

Parameter	OR	Standard error	*P* value	95% CI
*Central 1000 *μ*m region *				
Age	0.985	0.057	0.796	0.880–1.103
Drusen number (continuous)	1.297	0.195	0.083	0.966–1.740
Area occupied by drusen (continuous)	0.990	0.008	0.194	0.975–1.005
Pigmentation (yes/no)	0.148	0.183	0.123	0.013–1.673

*Central 3000 *μ*m region *				
Age	0.920	0.755	0.309	0.783–1.080
Drusen number (continuous)	1.091	0.033	**0.004**	1.028–1.158
Area occupied by drusen (continuous)	0.997	0.001	**0.021**	0.995–1.000
Pigmentation (yes/no)	0.018	0.031	**0.019**	0.001–0.512

*Central 6000 *μ*m region *				
Age	0.957	0.067	0.528	0.835–1.097
Drusen number (continuous)	1.028	0.013	**0.030**	1.003–1.054
Area occupied by drusen (continuous)	0.999	0.001	**0.034**	0.998–0.999
Pigmentation (yes/no)	0.036	0.053	**0.024**	0.002–0.649

**Table 6:** Model 2 of multivariate analysis: logistic regression with odds ratio (OR) estimates considering the totality of drusen.

Parameter	OR	Standard Error	*P* Value	95% CI
*Central 1000 *μ*m region *				
Age	1.006	0.040	0.871	0.931–1.088
Drusen number (continuous)	1.106	0.041	**0.007**	1.029–1.189
Area occupied by drusen (continuous)	0.996	0.003	0.200	0.990–1.002
Pigmentation (yes/no)	0.664	0.092	**0.049**	0.004–0.993

*Central 3000 *μ*m region *				
Age	0.949	0.071	0.480	0.820–1.098
Drusen number (continuous)	1.017	0.008	**0.024**	1.002–1.032
Area occupied by drusen (continuous)	0.999	0.001	0.205	0.998–1.001
Pigmentation (yes/no)	0.041	0.058	**0.024**	0.003–0.652

*Central 6000 *μ*m region *				
Age	0.962	0.068	0.580	0.837–1.105
Drusen number (continuous)	1.006	0.003	0.051	1.000–1.012
Area occupied by drusen (continuous)	0.995	0.000	0.134	0.999–1.000
Pigmentation (yes/no)	0.037	0.056	**0.029**	0.002–0.710

**Table 7:** Cumulative incidences of CNV on the fellow eyes of patients with unilateral exudative AMD between our and other studies.

Authors	Followup	*n*	Cumulative incidence (years)				
	(months)		2	3	4	5	10
**Marques et al.** (our study)	**60.9 (36–148)**	**89**	**23.6%**	**33.7%**	**38.2%**	**39.3%**	**47.2%**
Pauleikhoff et al. [24]	30.5 (6–80)	187	15.2%	26.1%	40.7%	—	—
Solomon et al. [25]	24–48	364	22%	—	37%	—	—
MPS [14]	60	228	12%	—	22%	42%	—
MPS [15]	60	670	19%	—	36%		—
van Leeuwen et al. [26]	48–156	35	—	—	—	38.7%	—
Uyama et al. [27]	47 (12–108)	170	5.6%	—	—	12.3%	—

## References

[1] Starr CE, Guyer DR, Yannuzzi LA (1998). Age-related macular degeneration: can we stem this worldwide public health crisis?. *Postgraduate Medicine*.

[2] Evans J (1996). Is the incidence of registrable age-related macular degeneration increasing?. *British Journal of Ophthalmology*.

[3] Klein R, Klein BEK, Linton KLP (1992). Prevalence of age-related maculopathy: the Beaver Dam Eye study. *Ophthalmology*.

[4] Klein R, Klein BEK, Knudtson MD, Meuer SM, Swift M, Gangnon RE (2007). Fifteen-year cumulative incidence of age-related macular degeneration. The Beaver Dam Eye study. *Ophthalmology*.

[5] Mukesh BN, Dimitrov PN, Leikin S (2004). Five-year incidence of age-related maculopathy: the visual impairment project. *Ophthalmology*.

[6] Oliver-Fernandez A, Bakal J, Segal S, Shah GK, Dugar A, Sharma S (2005). Progression of visual loss and time between initial assessment and treatment of wet age-related macular degeneration. *Canadian Journal of Ophthalmology*.

[7] Sivaprasad S, Membrey WL, Sivagnanavel V (2006). Second eye of patients with unilateral neovascular age-related macular degeneration: caucasians vs. Chinese. *Eye*.

[8] Klein R, Klein BEK, Jensen SC, Meuer SM (1997). The five-year incidence and progression of age-related maculopathy: the beaver dam eye study. *Ophthalmology*.

[9] Bressler NM, Bressler SB, Congdon NG (2003). Potential public health impact of age-related eye disease study results: AREDS Report No. 11. *Archives of Ophthalmology*.

[10] van Leeuwen R, Chakravarthy U, Vingerling JR (2003). Grading of age-related maculopathy for epidemiological studies: is digital imaging as good as 35-mm film?. *Ophthalmology*.

[11] Klein R, Meuer SM, Moss SE, Klein BEK, Neider MW, Reinke J (2004). Detection of age-related macular degeneration using a nonmydriatic digital camera and a standard film fundus camera. *Archives of Ophthalmology*.

[12] Bird AC, Bressler NM, Bressler SB (1995). An international classification and grading system for age-related maculopathy and age-related macular degeneration: the International ARM Epidemiological Study group. *Survey of Ophthalmology*.

[13] Clemons TE, Milton RC, Klien R, Seddon JM, Ferris FL (2005). Risk factors for the incidence of advanced age-related macular degeneration in the Age-Related Eye Disease study (AREDS): AREDS report no. 19. *Ophthalmology*.

[14] Macular Photocoagulation Study (MPS) (1993). Five-year follow-up of fellow eyes of patients with age-related macular degeneration and unilateral extrafoveal choroidal neovascularization. *Archives of Ophthalmology*.

[15] Macular Photocoagulation Study (MPS) (1997). Risk factors for choroidal neovascularization in the second eye of patients with juxtafoveal or subfoveal choroidal neovascularization secondary to age-related macular degeneration. *Archives of Ophthalmology*.

[16] Davis MD, Gangnon RE, Lee L (2005). The age-related eye disease study severity scale for age-related macular degeneration: AREDS report no. 17. *Archives of Ophthalmology*.

[17] Ferris FL, Davis MD, Clemons TE (2005). A simplified severity scale for age-related macular degeneration: AREDS report no. 18. *Archives of Ophthalmology*.

[18] Friberg TR, Bilonick RA, Brennen P (2012). Is drusen area really so important? An assessment of risk of conversion to neovascular amd based on computerized measurements of drusen. *Investigative Ophthalmology and Visual Science*.

[19] Friberg TR, Huang L, Palaiou M, Bremer R (2007). Computerized detection and measurement of drusen in age-related macular degeneration. *Ophthalmic Surgery Lasers and Imaging*.

[20] Silva R, Cachulo ML, Fonseca P (2011). Age-related macular degeneration and risk factors for the development of choroidal neovascularisation in the fellow eye: a 3-year follow-up study. *Ophthalmologica*.

[21] Friberg TR, Bilonick RA, Brennen PM (2011). Analysis of the relationship between drusen size and drusen area in eyes with age-related macular degeneration. *Ophthalmic Surgery Lasers and Imaging*.

[22] Scholl HPN, Dandekar SS, Peto T (2004). What is lost by digitizing stereoscopic fundus color slides for macular grading in age-related maculopathy and degeneration?. *Ophthalmology*.

[23] Landis JR, Koch GG (1977). The measurement of observer agreement for categorical data. *Biometrics*.

[24] Pauleikhoff D, Radermacher M, Spital G (2002). Visual prognosis of second eyes in patients with unilateral late exudative age-related macular degeneration. *Graefe’s Archive for Clinical and Experimental Ophthalmology*.

[25] Solomon SD, Jefferys JL, Hawkins BS, Bressler NM, Bressler SB (2007). Incident choroidal neovascularization in fellow eyes of patients with unilateral subfoveal choroidal neovascularization secondary to age-related macular degeneration: SST report no. 20 from the Submacular Surgery Trials Research Group. *Archives of Ophthalmology*.

[26] van Leeuwen R, Klaver CCW, Vingerling JR, Hofman A, De Jong PTVM (2003). The risk and natural course of age-related maculopathy: follow-up at 6 1/2 years in the Rotterdam study. *Archives of Ophthalmology*.

[27] Uyama M, Takeuchi M, Takahashi K (2000). The second eye of Japanese patients with unilateral exudative age related macular degeneration. *British Journal of Ophthalmology*.

[28] Friberg TR, Brennen PM, Freeman WR, Musch : DC (2009). Prophylactic treatment of age-related macular degeneration report number 2: 810-nanometer laser to eyes with drusen: bilaterally eligible patients. *Ophthalmic Surg Lasers Imaging*.

[29] Complications of Age-related Macular Degeneration Prevention Trial (CAPT) Research Group (2008). Risk factors for choroidal neovascularization and geographic atrophy in the complications of age-related macular degeneration prevention trial. *Ophthalmology*.

[30] Pieramici DJ, Bressler SB (1998). Age-related macular degeneration and risk factors for the development of choroidal neovascularization in the fellow eye. *Current Opinion in Ophthalmology*.

[31] Sandberg MA, Weiner A, Miller S, Gaudio AR (1998). High-risk characteristics of fellow eyes of patients with unilateral neovascular age-related macular degeneration. *Ophthalmology*.

[32] Chang B, Yannuzzi LA, Ladas ID, Guyer DR, Slakter JS, Sorenson JA (1995). Choroidal neovascularization in second eyes of patients with unilateral exudative age-related macular degeneration. *Ophthalmology*.

